# Impact of extracorporeal membrane oxygenation in immunocompetent children with severe adenovirus pneumonia

**DOI:** 10.1186/s12890-022-02284-5

**Published:** 2023-01-30

**Authors:** Tingting Shi, Chen Chen, Huifeng Fan, Minghua Yu, Ming Li, Diyuan Yang, Li Huang, Zhiqiang Nie, Gen Lu

**Affiliations:** 1 Department of Respiratory, Guangzhou Women and Children’s Medical Center, Guangzhou Medical University, No.9, Jinsui Road, Zhujiang New City, Tianhe District, Guangzhou, 510120 Guangdong China; 2 Pediatric Intensive Care Unit, Guangzhou Women and Children’s Medical Center, Guangzhou Medical University, Guangzhou, China; 3 Department of Cardiology, Guangzhou Women and Children’s Medical Center, Guangzhou Medical University, Guangzhou, China; 4 Department of Cardiology, Hypertension Research Laboratory, Guangdong Cardiovascular Institute, Guangdong Provincial People’s Hospital, Guangdong Academy of Medical Sciences, Guangzhou, China

**Keywords:** Extracorporeal membrane oxygenation, Children, Severe, Adenovirus, Pneumonia

## Abstract

**Background:**

Severe adenovirus (*Adv*.) pneumonia can cause significant mortality in young children. There has been no worldwide consensus on the impact of extracorporeal membrane oxygenation (ECMO) in immunocompetent children with severe *Adv.* pneumonia. This study aimed to assess the impact of ECMO in immunocompetent children with severe *Adv.* pneumonia.

**Methods:**

This study evaluated the medical records of 168 hospitalized children with severe *Adv*. pneumonia at the Guangzhou Women and Children’s Medical Center between 2019 and 2020.Nineteen patients in the ECMO group and 149 patients in the non-ECMO group were enrolled.

**Results:**

Between these two groups, there were no differences in host factors such as sex, age (all P > 0.05). Significant differences were observed in shortness of breath/increased work of breathing; cyanosis; seizures; tachycardia; the partial pressure of oxygen in arterial blood (PO_2_); the ratio of PaO_2_ to the fraction concentration of oxygen in inspired air (FiO_2_; P/F); white blood cell, lymphocyte, monocytes, lactate dehydrogenase (LDH), serum albumin, and procalcitonin levels; and, pulmonary consolidation (all P < 0.05). There were significant differences in the parameters of mechanical ventilation (MV) therapy and complications such as respiratory failure, acute respiratory distress syndrome, septic shock, length of hospitalization, and death (all P < 0.05). The maximum axillary temperatures, respiratory rates, heart rates and LDH levels after receiving ECMO were significantly lower than those before ECMO (all P < 0.05). Additionally, SPO_2_, PO_2_, and P/F were significantly higher than those before ECMO (all P < 0.05). In MV therapy, FiO_2_, PIP, and PEEP were significantly lower than those before ECMO (all P < 0.05).

**Conclusions:**

In our study, the clinical conditions of the patients in the ECMO group were much more severe than those in the non-ECMO group. Our study showed that ECMO might be beneficial for the patients with severe *Adv.* pneumonia.

## Background

Adenovirus (*Adv.*) is a common virus causing respiratory infection in different age groups (at least 5–10% of pediatric cases [1]). In immunocompromised persons, severe respiratory failure develops in 10–30% of cases [2, 3] and fatality rates for severe *Adv.* pneumonia may exceed 50% [4]. The clinical course of *Adv.* in immunocompetent patients is usually self-limiting [5]. However, deaths due to severe *Adv.* pneumonia have been described in previously healthy children [6], and some studies have shown that severe *Adv.* pneumonia can cause significant mortality in young children due to acute extensive pulmonary consolidation accompanying systemic multiple organ dysfunction syndrome (MODS) in the acute stage [7–9]. In the chronic stage, some children with severe *Adv.* pneumonia characterized by persistent wheezing develop necrotizing pneumonia, bronchiectasis, atelectasis, and bronchiolitis obliterans [10, 11].

No antiviral drug has been approved to treat *Adv* [3] and there are few prospective randomized controlled trials [12]. Children with severe *Adv.* infection should undergo bronchoscopy and be administered glucocorticoids and broad-spectrum antibiotics in the presence of bacterial coinfection and/or mechanical ventilation (MV) [5]. Although positive traditional medical therapies and mechanical ventilator support were administered, the conditions of some patients with severe *Adv.* pneumonia continued to deteriorate [5, 13]. Extracorporeal membrane oxygenation (ECMO) was first conducted in 1970 in infants with potentially reversible cardiac failure in whom maximal conventional ventilator treatment had failed [14]. Some retrospective studies showed that some neonatal, pediatric, and adult patients who needed ECMO for severe *Adv.* pneumonia had high hospital mortality (58–62%) [15, 16]. However, in recent years, some other studies have reported ECMO to be a potential effective support for severe *Adv.* pneumonia, and the administration of ECMO in these patients seemed to reduce the mortality (25.00–33.33%) [5, 13, 17].

Until now, there has been no worldwide consensus on the impact of ECMO in immunocompetent children with severe *Adv.* pneumonia. Here, we analyzed 168 pediatric patients with severe *Adv.* Pneumonia, including 19 in whom conventional treatments failed and ECMO were required in order to assess the impact of ECMO in immunocompetent children from a developing country.

## Materials and methods

### Objectives and data collection

This study included 19 patients with severe *Adv.* pneumonia requiring ECMO (ECMO group) who were admitted to the Guangzhou Women and Children’s Medical Center between January 2019 and December 2020. A group of 149 children with severe *Adv.* pneumonia who were hospitalized during the same period and did not receive ECMO was included as the control group (non-ECMO group). Children with inborn errors of immunity, hematologic malignancies, human immunodeficiency virus (HIV) infections, and comorbidities such as inherited metabolic diseases, neuromuscular diseases with immunocompromisation, and autoimmune diseases were excluded. Patients with incomplete data (discharged against medical advice or died within 24 h) were excluded. This study was approved by the Ethics Committee of the Guangzhou Women and Children’s Medical Center at Guangzhou Medical University. The study was performed in accordance with the ethical guidelines of the Declaration of Helsinki (7th revision).

For all patients, data on demographics; clinical characteristics; laboratory, microbiological, and radiological findings; complications; treatments including medication administered; MV treatments and life-support systems [continuous renal replacement therapy (CRRT), and ECMO]; and, outcomes were collected. MV data included the types of MV, the parameters of MV, and the ratio of the partial pressure of oxygen in arterial blood (PaO_2_) to the fraction concentration of oxygen in inspired air (FiO_2_; P/F). ECMO group data included changes in the various indices before and after ECMO, such as clinical characteristics, laboratory findings, the parameters of MV therapy, and P/F.

### Diagnostic methods and setting

A list of patients was generated by identifying nasopharyngeal secretions positive for *Adv.* using polymerase chain reaction. Blood, sputum, and/or bronchoalveolar lavage cultures were obtained for suspected bacterial, *Mycoplasma pneumoniae*, and fungal infections. All the patients underwent viral testing of nasopharyngeal secretions by indirect immunofluorescence during the acute stage to identify other respiratory virus infections. All the patients underwent chest radiography, and some underwent high-resolution computed tomography (HRCT) due to the presence of a wide range of lesions on chest radiography. Additionally, flexible bronchoscopy was performed in patients who exhibited a wide range of lesions on HRCT. All the patients underwent echocardiography to rule out heart disease and to indirectly determine the pulmonary artery pressure. A serum-specific antibody test to rule out HIV infection was conducted, and all the patients tested negative for HIV.

The criteria for severe pneumonia were defined according to the British Thoracic Society guidelines [18]. There is no worldwide consensus on the clinical conditions for which ECMO should be conducted. In our study, ECMO was considered in the patients in whom positive traditional medical therapies and MV support did not seem to work, according to the following suggestions of the Organization for Extracorporeal Life Support (ELSO) [19]: (1) Severe respiratory failure (PaO_2_/FiO_2_ ratio < 60–80 or Oxygenation index (OI) > 40; (2) lack of response to MV and other associated therapies (prone position, inhaled nitric oxide, high-frequency oscillatory ventilation [HFOV]); and, (3) high MV pressures. Acute respiratory distress syndrome (ARDS) was defined using the pediatric acute lung injury consensus conference criteria and all the patients before ECMO initiation were treated with standard care according to these criteria [20]. Septic shock was defined using the International Pediatric Sepsis Consensus Conference criteria [21].

### Statistical analyses

Categorical data were presented as frequencies with the corresponding percentages, and continuous data were presented as mean ± standard deviation (x ± s). The χ^2^ or Fisher exact test was used to determine the associations between the ECMO and non-ECMO groups in the categorical variables. The Mann–Whitney test was used to measure the changes in various indices after administering ECMO. The significance level of all tests was set at p < 0.05. For all statistical analyses, R Version 3.6.1 software was utilized.

## Results

### Demographics and clinical characteristics

**Fig. 1:**
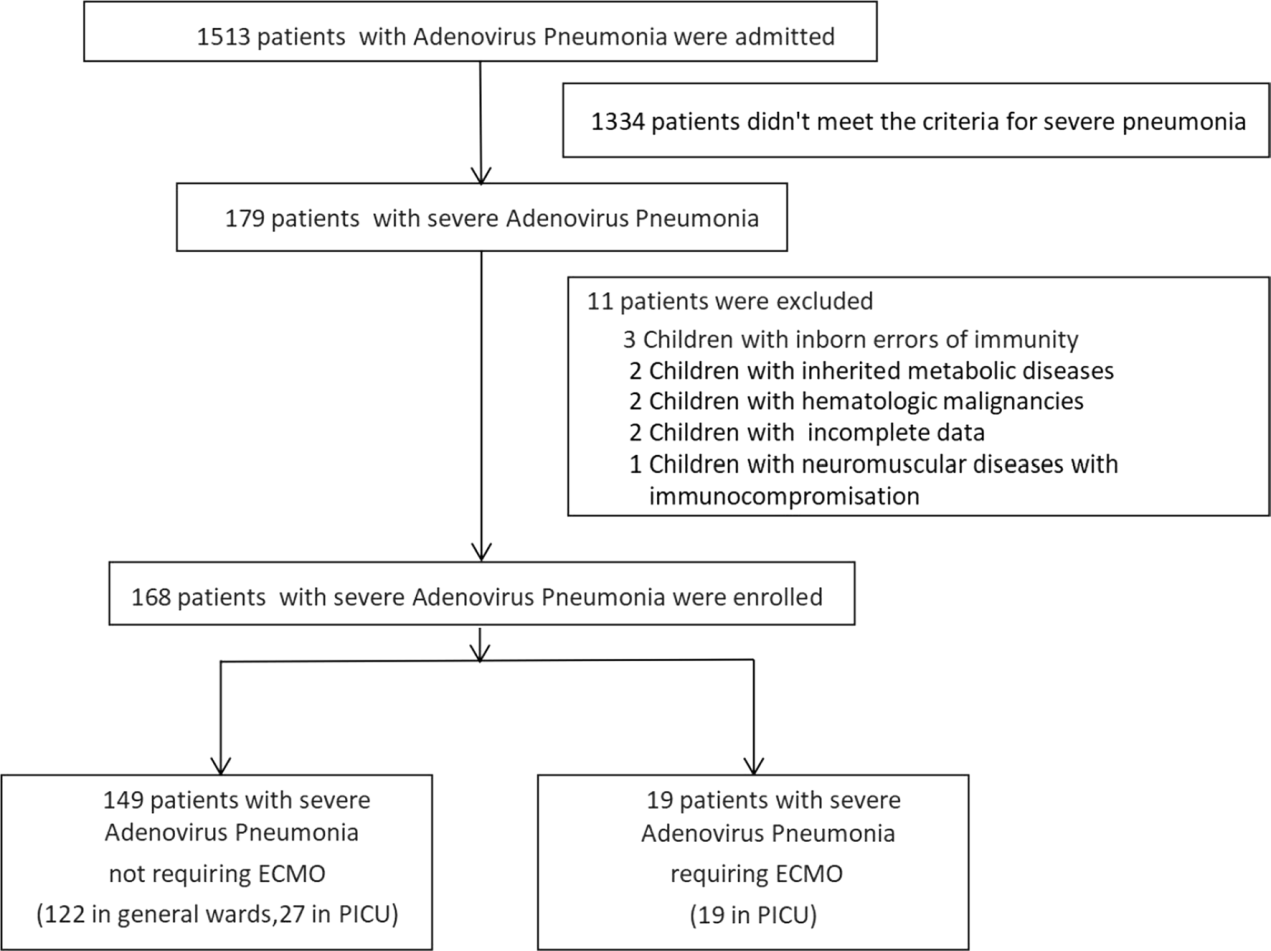
The flowchart of the patients enrollments

In the 2-year period, the nasopharyngeal secretions of 179 hospitalized children with severe pneumonia tested positive for *Adv*. Eleven patients were excluded based on the exclusion criteria. Figure 1 showed the flowchart of the patients enrollments. The demographics and clinical characteristics of the 168 hospitalized patients with severe *Adv.* pneumonia, including 19 patients in the ECMO group and 149 patients in non-ECMO group, are presented in Table 1. Of these, 60.7% (102/168) were boys and 39.3% (66/168) were girls. The mean age was 30.79 ± 27.88 months, with ages ranging from 1 to 144 months. Fever and cough (100%, 168/168) were the most common symptoms in our study, followed by cyanosis and shortness of breath respectively (20.24%, 34/168). The mean fever duration was 13.92 ± 6.82 days. The mean oxygen saturation (SPO_2_) was 89.92 ± 13.14%. Crackles (87.50%, 147/168) was the most common physical examination finding in our study, followed by tachypnea (respiratory rate > 70/min [≤ 1 year] or > 60/min [> 1 year]) (32.14%, 54/168), and tachycardia (heart rate > 180/min [≤ 1 year] or > 160/min [> 1 year]) (38.69%, 65/168).

**Table 1:** Demographics and Clinical characteristics of 168 hospitalized children with severe *Adv.* pneumonia

Characteristics	Total	non-ECMO group	ECMO group	P-value
	N = 168 (n, %) (x ± s)	N = 149 (n, %) (x ± s)	N = 19 (n, %) (x ± s)	
*Demographics*				
Male gender(n)	102 (60.71%)	89 (59.53%)	13 (68.42%)	0.46
Age (months)	30.79 ± 27.88	31.52 ± 28.36	25.0 ± 24.0	0.32
*Clinical characteristics*				
Clinical symptoms				
Fever (n)	168 (100%)	149 (100%)	19 (100%)	1.00
Fever (days)	13.92 ± 6.82	14.16 ± 6.81	11.94 ± 6.78	0.11
Cough (n)	168 (100%)	149 (100%)	19 (100%)	1.00
Shortness of breath (n)	34 (20.24%)	19 (12.75%)	15 (78.95%)	**0.00**
Cyanosis (n)	34 (20.24%)	19 (12.75%)	15 (78.95%)	**0.00**
Vomiting/diarrhea (n)	34 (20.24%)	30 (20.13%)	4 (21.05%)	0.93
Seizures (n)	7 (4.17%)	3 (2.01%)	4 (21.05%)	**0.00**
Physical exam findings				
Respiratory rate > 70/min (≤ 1 years) or > 60/min (> 1 year) (n)	54 (32.14%)	35 (23.49%)	19 (100%)	**0.00**
Heart rate > 180/min (≤ 1 years) or > 160/min (> 1 year) (n)	65 (38.69%)	51 (34.23%)	14 (73.68%)	**0.00**
Oxygen saturation (%)	89.92 ± 13.14	92.86 ± 20.17	66.84 ± 20.17	**0.00**
Crackles (n)	147 (87.50%)	130 (87.25%)	17 (89.47%)	0.78
Wheezing (n)	34 (20.24%)	28 (18.79%)	6 (31.58%)	0.19
CRT > 3 s (n)	18 (10.71%)	8 (5.37%)	10 (52.63%)	**0.00**

*P* < 0.05 are shown in bold

*CRT* capillary refilling time

### Laboratory, radiological, and microbiological findings

**Fig. 2:**
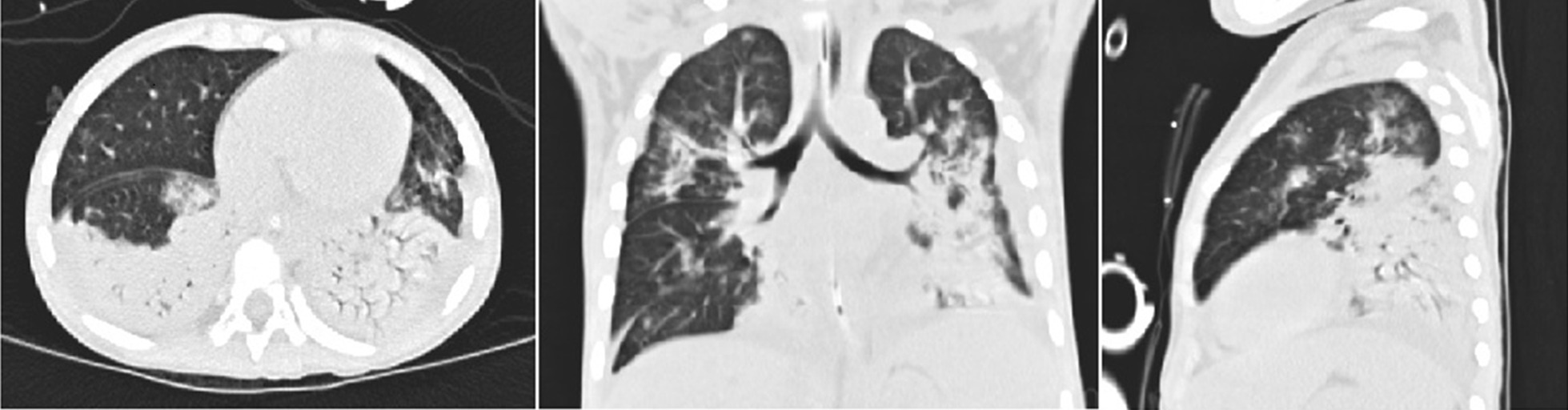
High-resolution CT scan of the chest revealing areas of airspace consolidation in bilateral lower lobes in a 3-year-old child with severe adenovirus (*Adv.*) pneumonia

The abnormal laboratory, radiological, and microbiological findings are shown in Table 2. In the 168 patients, the mean PO_2_ was 10.01 ± 2.62 kPa, and the mean P/F was 225.21 ± 97.89. The mean white blood cell (WBC) count was 8.09 ± 5.14 × 10^9^/L, and the mean procalcitonin (PCT) level was 5.68 ± 15.85 ng/L. The chest scans revealed diffuse infiltrations in both lungs in most patients; and, some cases exhibited segmental consolidation (Fig. 2), especially in the ECMO group (P < 0.05). The other main radiographic finding was pleural effusion (50.60%, 85/168). Among the 168 patients, besides *Adv.*, another causative agent (defined as coinfection) was detected, including *M. pneumoniae* coinfections in 50.60% (85/168), bacterial coinfections in 21.43% (36/168), and other viral coinfections in 17.85% (30/168). In the bacterial coinfection cases, *Haemophilus influenzae* (7.74%,13/168) was the most common bacterium isolated from patients with *Adv.* infection, followed by *Klebsiella pneumoniae* (4.12%, 7/168). The most common viruses isolated were respiratory syncytial virus and parainfluenza, respectively (6.55%, 11/168).

**Table 2:** The laboratory, radiological findings, and pathogenies of 168 hospitalized children with severe *Adv.* pneumonia

Characteristics	Total	non-ECMO group	ECMO group	P-value
	N = 168 (n, %) (x ± s)	N = 149 (n, %) (x ± s)	N = 19 (n, %) (x ± s)	
*Laboratory index*				
PO_2_ (kPa)	10.01 ± 2.62	10.25 ± 2.46	8.12 ± 3.11	**0.00**
PCO_2_ (kPa)	4.92 ± 1.02	4.85 ± 0.75	5.42 ± 2.18	0.47
P/F	225.21 ± 97.89	246.12 ± 82.8	61.26 ± 23.33	**0.00**
WBC (× 10^9/L)	8.09 ± 5.14	8.53 ± 5.2	4.66 ± 2.88	**0.00**
Monocyte (× 10^9/L)	0.48 ± 0.51	0.51 ± 0.53	0.22 ± 0.19	**0.00**
Lymphocyte (× 10^9/L)	2.58 ± 1.85	2.73 ± 1.88	1.41 ± 0.96	**0.00**
Hemoglobin (g/L)	103.89 ± 16.52	105.26 ± 15.93	93.21 ± 17.58	**0.00**
Lactate dehydrogenase (U/L)	1084.54 ± 879.99	965.91 ± 823.25	2014.78 ± 762.96	**0.00**
Serum albumin (g/L)	32.55 ± 5.96	33.08 ± 5.83	28.42 ± 5.44	**0.00**
C-reactive protein (mg/L)	32.80 ± 39.31	33.90 ± 41.12	24.17 ± 18.62	0.98
PCT (ng/ml)	5.68 ± 15.85	5.38 ± 16.12	8.06 ± 13.66	**0.01**
*Radiological finding (s)*				
X-ray/CT				
Consolidation	97 (57.73%)	82 (53.03%)	15 (78.95%)	**0.04**
Pleural effusion	85 (50.60%)	73 (48.99%)	12 (63.16%)	0.24
Pneumothorax	6 (3.57%)	4 (5.37%)	2 (10.53%)	0.08
*Co-infections (n)*				
Adenovirus-Virus	30 (17.86%)	24 (14.29%)	6 (31.58%)	0.10
Respiratory syncytial virus	11 (6.55%)	8 (5.37%)	3 (15.79%)	0.08
Parainfluenza	11 (6.55%)	8 (5.37%)	3 (15.79%)	0.08
FA/FB	4 (23.81%)	4 (2.68%)	0 (0.00%)	0.47
Rhinovirus	4 (23.81%)	4 (2.68%)	0 (0.00%)	0.47
Adenovirus-Bacteria	36 (21.43%)	29 (19.46%)	7 (36.84%)	0.08
Haemophilus influenzae	13 (77.38%)	12 (8.05%)	1 (5.26%)	0.67
Klebsiella pneumoniae	7 (41.67%)	6 (4.03%)	1 (5.26%)	0.80
Staphylococcus aureus	6 (35.71%)	4 (2.68%)	2 (10.53%)	0.08
Acinetobacter baumannii	3 (17.86%)	2 (1.34%)	1 (5.26%)	0.22
Pseudomonas aeruginosa	5 (29.76%)	4 (2.68%)	1 (5.26%)	0.53
Moraxella catarrhalis	2 (1.19%)	1 (0.76%)	1 (5.26%)	0.08
Adenovirus-Mycoplasma pneumoniae	85 (50.60%)	79 (53.02%)	6 (31.58%)	0.07

*P* < 0.05 are shown in bold

### Treatment, complications, and outcome

Table 3 shows the patients’ treatments. Of the 168 patients, 87.88% (145/168) received antibiotic therapy; 86.43% (162/168), immunoglobulin; and, 64.88% (109/168), corticosteroids; and, 24.40% (41/168), assisted ventilation, including 11.31% (19/168) in the ECMO group and 13.10% (22/168) in the non-ECMO group. In the MV therapy before receiving ECMO, 22.02% (37/168) patients had FiO_2_ > 60%, 16.07% (27/168) patients had peak inspiratory pressure (PIP) > 30 cmH_2_O, 16.07% (27/168) patients had end-expiratory pressure (PEEP) > 8 cmH_2_O, and 8.93% (15/168) of the patients received CRRT therapy.

**Table 3:** Treatments and outcomes of 168 hospitalized children with severe *Adv.* pneumonia

Characteristics	Total	non-ECMO group	ECMO group	P-value
	N = 168 (n, %) (x ± s)	N = 149 (n, %) (x ± s)	N = 19 (n, %) (x ± s)	
*Treatments (n)*				
Antibiotics	145 (97.32%)	126 (84.53%)	19 (100.00%)	0.65
Immunoglobulin	162 (96.43%)	145 (97.32%)	17 (89.47%)	0.08
Corticosteroid	109 (64.88%)	96 (64.43%)	13 (68.42%)	0.69
*Mechanical ventilation (n)*				
Conventional Mechanical ventilation	41 (24.40%)	22 (14.77%)	19 (100.00%)	**0.00**
High-frequency Oscillatory Ventilation	19 (11.31%)	4 (2.68%)	15 (78.95%)	**0.00**
FiO_2_ > 60%	37 (22.02%)	18 (12.08%)	19 (100.00%)	**0.00**
PIP > 30cmH_2_O	27 (16.07%)	15 (10.07%)	12 (63.16%)	**0.00**
PEEP > 8cmH_2_O	27 (16.07%)	10 (6.71%)	16 (84.21%)	**0.00**
*CRRT*	15 (8.93%)	3 (2.01%)	12 (63.16%)	**0.00**
*Complication*				
Respiratory failure	47 (27.98%)	28 (18.79%)	19 (100.00%)	**0.00**
ARDS	18 (10.71%)	3 (2.01%)	15 (78.95%)	**0.00**
Septic shock	18 (10.71%)	4 (2.68%)	14 (73.68%)	**0.00**
Acute renal failure	4 (2.38%)	0 (0.00%)	4 (21.05%)	**0.00**
Pneumorrhagia	3 (1.79%)	1 (0.67%)	2 (10.53%)	**0.00**
Gastrointestinal hemorrhage	3 (1.79%)	2 (1.19%)	1 (5.26%)	0.22
Heart failure	2 (1.19%)	0 (0.00%)	2 (10.53%)	**0.00**
Liver failure	2 (1.19%)	0 (0.00%)	2 (10.53%)	**0.00**
*Outcomes*				
The length of hospitalization (days)	18.12 ± 13.08	15.82 ± 8.31	37.13 ± 25.31	**0.00**
Dead (n)	13 (7.74%)	6 (3.57%)	7 (36.84%)	**0.00**

*P* < 0.05 are shown in bold

In the 168 patients with severe *Adv.* pneumonia, the most common complication was respiratory failure (27.98%, 47/168), followed by ARDS and septic shock respectively (10.71%, 18/168). 39 patients (23.21%, 39/168) admitted to pediatric intensive care unit (PICU), including 20 patients in non-ECMO group and 19 in ECMO group. The mean length of ECMO was 10.26 ± 7.38 days (range, 2 to 36 days), and the mean length of hospitalization was 18.12 ± 13.08 days. Among all patients, seven died in the ECMO group (4.17%, 7/168). The mortality rate in the ECMO group was 36.84% (7/19).

### Comparisons of the characteristics between the ECMO and non-ECMO groups

Comparisons of the characteristics between the ECMO and non-ECMO groups are shown in Tables 1, 2, and 3. Table 1 shows the demographic and clinical characteristics of the patients. Between these two groups, there were no differences in host factors such as sex, age (all P > 0.05). However, there were significant differences in some clinical characteristics, including shortness of breath/increased work of breathing, cyanosis, seizures, tachycardia, SPO_2_, and capillary refilling time (CRT) > 3S (all P < 0.05). Table 2 presents the laboratory, radiological, and microbiological findings of the patients. Significant differences were observed in some laboratory and radiological findings, including PO_2_; P/F; WBC, lymphocyte, monocyte, lactate dehydrogenase (LDH), serum albumin (ALB), and PCT levels; and, pulmonary consolidation (all P < 0.05). There were no differences in viral, bacterial, *Mycoplasma pneumoniae*, and fungal coinfections between these two groups (all P > 0.05). Table 3 shows that the significant differences in the treatment and complications before ECMO between the ECMO and non-ECMO groups were in the parameters of MV therapy and complications such as respiratory failure, ARDS, and septic shock (all P < 0.05). There were significant differences in the length of hospitalization and death (all P < 0.05).

### Comparisons of various indices of patients with severe *Adv.* pneumonia after receiving ECMO

**Fig. 3:**
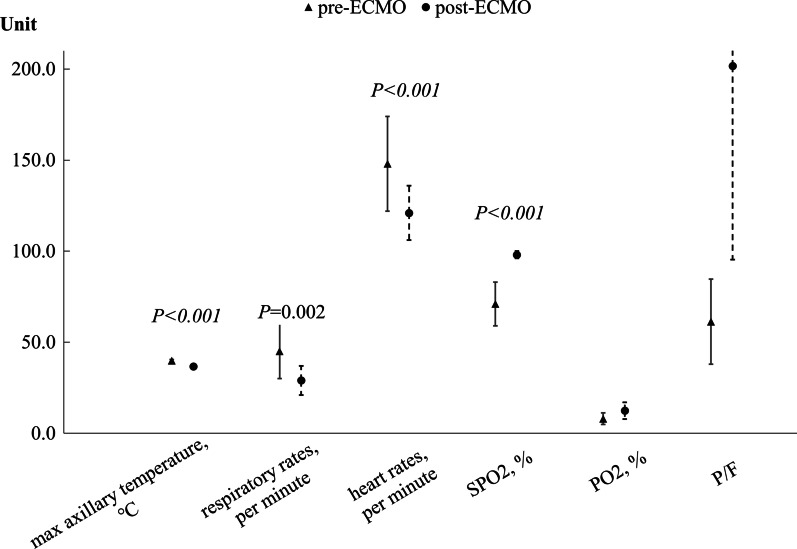
Comparisons of various indexes of clinical characteristics and laboratory findings of severe *Adv.* pneumonia patients after receiving ECMO, including the maximum axillary temperatures, respiratory rates, heart rates, SPO_2_, PO_2_, and P/F (all P < 0.05). LDH differed greatly in dimension from other parameters, so it was not listed in the Fig. 2. The mean LDH before ECMO was (2015.00 ± 763.00)U/L, the mean LDH after ECMO was (1061.00 ± 875.00)U/L, and there was significant difference (P = 0.00)

**Fig. 4:**
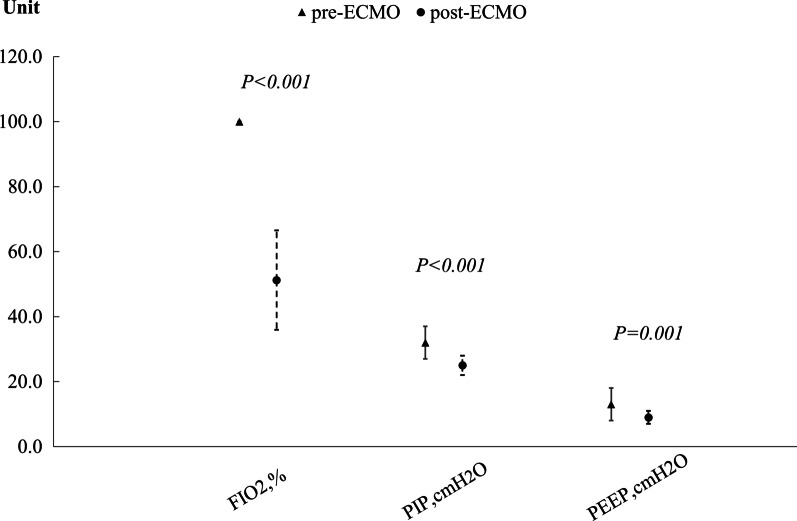
Comparisons of various indexes of MV therapy of severe *Adv.* pneumonia patients after receiving ECMO, including FiO_2_, PIP, and PEEP (all P < 0.05)

Table 4 showed the detail characteristics of 19 patients with severe *Adv.* Pneumonia receiving ECMO.The results of the self pre and post control observation in ECMO group were presented below.In terms of clinical characteristics and laboratory findings, the maximum axillary temperatures, respiratory rates, heart rates, and LDH levels after receiving ECMO were significantly lower than those before ECMO (all P < 0.05; Fig. 3). Additionally, SPO_2_, PO_2_, and P/F were significantly higher than those before ECMO (all P < 0.05; Fig. 3). In MV therapy, FiO_2_, PIP, and PEEP were significantly lower than those before ECMO (all P < 0.05; Fig. 4).

**Table 4:** Detail characteristics of 19 hospitalized children with severe Adv. Pneumonia receiving ECMO

No.	Duration of IMV (days)	Duration from onset of illness to ECMO (days)	Duration of ECMO (days)	Duration of ICU (days)	Duration of hospitalization (days)	Main complications	Pre-ECMO arterial blood gas analysis				Pre-ECMO weaning arterial blood gas analysis				Post ECMO weaning arterial blood gas analysis				Outcomes
							PaO_2_ (kPa)	PaCO_2_ (kPa)	FiO_2_	P/F	PaO_2_ (kPa)	PaCO_2_ (kPa)	FiO_2_	P/F	PaO_2_ (kPa)	PaCO_2_ (kPa)	FiO_2_	P/F	
1	12	22	7	15	26	ARDS,Septic shock	11.50	4.00	100%	86.25	24.00	5.60	40%	450.00	20.00	5.90	50%	300.00	Survival
2	19	23	7	23	38	ARDS,Septic shock	7.60	4.10	100%	57.00	14.30	4.30	40%	268.13	13.30	4.90	50%	199.50	Survival
3	90	26	12	93	93	Pneumothorax,ARDS,Septic shock	7.16	4.96	100%	53.70	12.80	6.10	70%	137.14	NA	NA	NA	NA	**Death**
4	27	21	12	45	71	ARDS,Septic shock	6.90	5.10	100%	51.75	8.50	5.30	90%	70.83	10.90	5.50	70%	116.79	Survival
5	13	11	4	17	21	ARDS,Septic shock	7.50	5.90	90%	62.50	11.40	4.60	45%	190.00	12.30	4.90	50%	184.50	Survival
6	26	35	13	39	39	ARDS,Septic shock	9,6	4.70	100%	72.00	10.40	8.00	30%	260.00	8.40	7.30	60%	105.00	**Death**
7	7	18	3	14	27	ARDS,Septic shock	4.90	5.50	100%	36.75	12.40	4.60	50%	186.00	13.00	5.90	55%	177.27	Survival
8	14	9	8	27	46	Pneumothorax,ARDS,Septic shock	5.70	6.70	100%	42.75	18.30	5.90	55%	249.55	16.30	5.00	50%	244.50	Survival
10	18	16	11	23	28	ARDS,Septic shock	8.70	5.30	100%	65.25	14.90	5.50	48%	232.81	13.20	4.90	45%	220.00	Survival
11	7	10	6	7	7	Septic shock	5.20	7.90	100%	39.00	16.10	5.90	30%	402.50	NA	NA	NA	NA	**Death**
12	14	10	13	14	14	Acute renal failure, Pneumorrhagia, Gastrointestinal hemorrhage	4.10	5.50	100%	30.75	7.20	6.10	50%	108.00	8.20	7.10	60%	102.50	**Death**
13	6	8	3	6	6	Pleural effusion, ARDS, Septic shock,Liver failure,Acute renal failure	2.10	13.20	100%	15.75	5.50	6.80	50%	82.50	6.20	6.50	65%	71.54	**Death**
14	10	9	4	17	23	Pleural effusion,Septic shock	7.50	4.50	100%	56.25	9.80	5.20	45%	163.33	11.90	4.80	50%	178.50	Survival
15	17	7	10	24	29	Pleural effusion, ARDS,	8.90	3.20	100%	66.75	17.70	7.60	45%	295.00	17.70	6.50	60%	221.25	Survival
16	17	10	8	25	31	Pleural effusion, ARDS,Pneumorrhagia	10.70	3.60	100%	80.25	12.90	4.40	60%	161.25	12.90	4.50	55%	175.91	Survival
17	42	16	36	45	45	Pleural effusion, ARDS,Acute renal failure	9.89	4.46	100%	74.18	7.30	6.40	55%	99.55	6.30	7.80	60%	78.75	**Death**
18	28	13	16	33	33	Pleural effusion, ARDS,Septic shock Heart failure,Liver failure, Acute renal failure	15.90	4.90	100%	119.25	8.70	4.40	80%	81.56	7.20	6.80	80%	67.50	**Death**
19	18	31	9	28	46	ARDS,Septic shock	10.40	5.60	100%	78.00	14.00	5.90	40%	262.50	13.10	5.70	50%	196.50	Survival

*P* < 0.05 are shown in bold

*NA* Patients NO.3 and NO.11 died before ECMO weaning because of some severe ECMO complications

## Discussion

Adenovirus (*Adv.*) infection is self-limiting in the majority of the immunocompetent population, but some immunocompetent children with *Adv.* infection develop severe pneumonia, myocarditis, hepatitis, encephalitis, etc. [8], which may quickly lead to refractory respiratory failure/hypoxemia, ARDS, MODS, and even death. In our two-year study, of the 19 patients with severe *Adv.* pneumonia undergoing ECMO, 100% of the patients developed severe complications, the most common of which was severe respiratory failure. In our seven patients who died in the ECMO group, the main causes of death were refractory hypoxic respiratory failure, ARDS, refractory septic shock, and MODS, which is in line with the previous literature [8, 22]. When severe *Adv.* pneumonia progressed into MODS, the mortality rate was higher than 50% [22]. In our study, the patients in the ECMO group who had more complications such as ARDS and MODS accounted for most of the deaths.

Moreover, we compared the characteristics of the patients between the ECMO and non-ECMO groups, and there were no differences in the demographics. Several findings were noteworthy: (1) the clinical conditions of the patients in the ECMO group, such as shortness of breath, cyanosis, seizures, and tachycardia, were more severe than those in the non-ECMO group, even with positive traditional medical therapies and high-parameter MV support (FiO_2_ > 60%, PIP > 30 cmH_2_O, PEEP > 8 cmH_2_O); (2) there were noticeably increased levels of PCT and LDH and noticeably decreased levels of WBC, lymphocytes, and ALB, which might be risk factors for severe *Adv.* pneumonia requiring ECMO support, which may induce refractory hypoxic respiratory failure, ARDS, refractory septic shock, and MODS, and; (3) we found that the monocyte levels in the ECMO group were significantly lower than those in the non-ECMO group, which may be a predictor of respiratory failure, in line with a previous study [23]. This may be because the *Adv.* infection initially induces cytokine secretion that may contribute to monocyte infiltration during the disease process. *Adv.* pneumonia is more severe, and monocyte chemotaxis or inflammatory cell production can become uncontrolled or aberrant [23, 24].

ARDS is a severe pulmonary inflammatory process accompanied by alveolar damage and hypoxemic respiratory failure [25]. In our study, 15 patients in the ECMO group who showed more severe consolidation in the radiological findings progressed to ARDS (P < 0.05). For patients with refractory hypoxic respiratory failure/ARDS in the ECMO group, the P/F before the initiation of ECMO was extremely low (61.26 ± 23.33). The P/F was statistically different between the ECMO and non-ECMO groups (P < 0.05), which meant that the patients in the ECMO group were more likely to develop ARDS or even pneumorrhagia. MV therapy remains the mainstay of management in most patients with severe *Adv.* Pneumonia [5], along with conventional MV and HFOV. In our study, most of the patients in the ECMO group had high MV parameters (FiO_2_ > 60%, PIP > 30 cmH_2_O, and PEEP > 8 cmH_2_O), and there was a statistical difference between the two groups (P < 0.05). However, the treatment did not seem to work in the ECMO group in our study, and the conditions of the patients continued to deteriorate, despite positive traditional medical therapies, CRRT, and MV support.

Previous studies [5, 13, 15–17] have controversial opinions on the impact of ECMO in children with severe *Adv.* pneumonia. Various factors affected mortality in patients undergoing ECMO [26–28], such as the condition of the patient, the time of undergoing ECMO, the duration of ECMO, and the complications of ECMO. In our study, by comparing patients' data before and after receiving ECMO, we found that ECMO significantly improved the conditions of the patients, including clinical characteristics, laboratory findings, and MV parameters. After receiving ECMO, the maximum axillary temperatures, respiratory rates, and heart rates of the patients on ECMO were significantly lower than those before receiving ECMO. As in the case of patients with the novel coronavirus disease (COVID-19) pneumonia [29], serum LDH was significantly elevated in the ECMO group in our study. LDH is released during tissue damage and is involved in various pathophysiological processes. Various disorders can increase LDH levels, such as infectious disease, heart failure, hypothyroidism, and cancer [29, 30]. The inflammatory response is a nonspecific response to hypoxia, tissue injury, and necrosis [31]. The elevated LDH in the ECMO group in our study may indicate that the serious pulmonary damage caused by *Adv.* led to hypoxia, tissue injury, septic shock, and other organ damage. During our study, we found that the serum LDH level was significantly lower than that before receiving ECMO, which indicated that ECMO could reduce the inflammatory response in severe *Adv.* pneumonia. Previous studies [32, 33] have suggested that the primary benefit of ECMO was providing rest to the lungs from high pressure and FiO_2_ ventilation, thus minimizing ventilator-induced lung injury, to overcome intractable hypoxemia or even ARDS. Our present results showed that SPO_2_, PO_2_, and P/F were significantly higher than those before ECMO, and FiO_2_, PIP, and PEEP were significantly lower than those before ECMO. SPO_2_, PO_2_, P/F, and MV parameters improved significantly in more than half of the patients in the ECMO group with intractable hypoxemia or ARDS. ECMO provided the lungs with sufficient rest, which could successfully preserve the pulmonary function from progressing to ARDS and help the patient recover from the severe pulmonary damage caused by *Adv*. According to previous retrospective studies [15, 16], the mortality of patients requiring ECMO for severe *Adv.* pneumonia was 58–62%. In our study, compared with the study above, the mortality rate in the ECMO group was much lower (36.8%, 7/19). The reason might be that below: first, the development of ECMO technical applications, which can reduce the occurrences of severe complications caused by ECMO; second, the standard care of ARDS before ECMO initiation, and third, standardized use and management of ECMO. All of these reasons above can reduce the mortality rate. Meanwhile, we found that our low mortality rate was familiar with recent study (33.33%) [5]. The results of our comparisons of various indices of patients with severe *Adv.* pneumonia after receiving ECMO and their relatively low mortality indicated that ECMO was beneficial for patients with severe *Adv.* pneumonia.

Several common complications associated with ECMO occurred in our study, such as bleeding, circuit clotting, aeroembolism, hemodynamic instability, neurological dysfunction, acute renal insufficiency, and technical errors, which were consistent with previous reports [27, 28]. In our study, 2 patients died because of severe ECMO complications. The causes of death were severe thromboembolism and neurological dysfunction (cerebral infarction).

Our study has several limitations. First, inflammatory markers, such as ferritin and cytokines, which can reflect inflammatory storm changes in patients, were not assessed or dynamically observed. Another limitation of our study is the lack of attention on the specific serotyping of *Adv.*, the differences in which could affect the severity of the *Adv.* infection. Third, immunocompromised and those excluded patients were not included in present stud, multivariate analysis can be performed in the future study. Finally, our study was a single-center retrospective study, and the number of ECMO samples was small. A large prospective investigation is urgently needed to confirm the impact and critical time of ECMO, and to improve the management of ECMO complications in severe *Adv.* pneumonia in order to decrease its mortality.

## Conclusions

In our study, the clinical conditions of the patients in the ECMO group were much more severe than those in the non-ECMO group. The max axillary temperatures, respiratory rates, heart rates, LDH levels, FiO_2_, PIP, and PEEP were significantly lower than those before ECMO, and SPO_2_, PO_2_, and P/F were significantly higher than those before ECMO. According to the results of the self pre and post control observation in ECMO group and along with the relatively low mortality in the ECMO group, our study showed that the ECMO might be beneficial for the patients with severe *Adv.* pneumonia.

## Data Availability

The datasets used and/or analyzed during the current study are available from the corresponding author on reasonable request.

## Abbreviations

*Adv.*: Adenovirus
ALB: Serum albumin
ARDS: Acute Respiratory Distress Syndrome
COVID-19: Novel coronavirus disease. CRRT: Continuous Renal Replacement Therapy
CRT: Capillary Filling Time
ECMO: Extracorporeal Membrane Oxygenation
ELSO: Organization for Extracorporeal Life Support
FiO2: The fraction concentration of oxygen in inspired air
HIV: Human immunodeficiency virus
HFOV: High-frequency oscillatory ventilation
HRCT: High-resolution Computed Tomography
LDH: Lactate dehydrogenase
MODS: Multiple organ dysfunction syndrome
MV: Mechanical ventilation
OI: Oxygenation index
PaO2: Partial pressure of oxygen in arterial blood
PCT: Procalcitonin
PEEP: End-expiratory pressure
P/F: The ratio of the partial pressure of oxygen in arterial blood to the fraction concentration of oxygen in inspired air
PICU: Pediatric intensive care unit
PIP: Peak inspiratory pressure
SPO2: Oxygen saturation
WBC: White blood cell
